# Specialized Rehabilitation Centers (CER) in the SUS and the impact of the covid-19 pandemic

**DOI:** 10.11606/s1518-8787.2023057004807

**Published:** 2023-05-11

**Authors:** Bianca Tomi Rocha Suda, Paulo Henrique dos Santos Mota, Aylene Bousquat

**Affiliations:** I Universidade de São Paulo Faculdade de Saúde Pública Programa de Pós-Graduação em Saúde Pública São Paulo SP Brasil Universidade de São Paulo. Faculdade de Saúde Pública. Programa de Pós-Graduação em Saúde Pública. São Paulo, SP, Brasil; II Universidade de São Paulo Faculdade de Saúde Pública Departamento de Política, Gestão e Saúde São Paulo SP Brasil Universidade de São Paulo. Faculdade de Saúde Pública. Departamento de Política, Gestão e Saúde. São Paulo, SP, Brasil

**Keywords:** Rehabilitation Centers, Unified Health System, Health Services Accessibility, COVID-19

## Abstract

**OBJECTIVE:**

To analyze the impact of the covid-19 pandemic on the functioning of Specialized Rehabilitation Centers (CER) in the SUS.

**METHODS:**

An analysis of the variation in outpatient production of the CER was carried out based on data from the Outpatient Information System of the Unified Health System (SIA-SUS) from March 2019 to December 2021. Such results were compared with CER managers’ perceptions about the impacts of the pandemic on the units, measured by a web survey applied between November 2020 and February 2021. Monthly averages of 247 procedures were calculated, organized into 18 groups, for three periods – year before the pandemic (YBP) and first (YP1) and second (YP2) years of the pandemic. Through the online form, information was collected on: operation and organization of services; post-covid-19 rehabilitation; actions to support the needs of users and professionals; strategies and challenges experienced.

**RESULTS:**

There was a 33.3% reduction in the total number of procedures in YP1 compared to YBP. There were no reductions in procedures performed by nurses and for ostomates. There was greater impairment for group activities, visual therapies and home visits. In YP2, there was a recovery of averages in relation to YBP in 11 groups of procedures, with an increase of 104.1% in Cardiorespiratory Physiotherapy. In the answers to the online form, 91.7% of the managers indicate structural and/or organizational changes in the CER, such as: creation of prioritization criteria for assistance; introduction of telerehabilitation; changes in the work process and; provision of professional training. Half of the CER already treated people with covid-19 sequelae, but not all of them had been trained to do so. Limitations in participation and social support for PWD were identified.

**CONCLUSIONS:**

There was a severe impact of the covid-19 pandemic on the CER. Added to the damming up of previous demands are those of post-covid-19 users, configuring a challenging picture. It is necessary to strengthen the Care Network for Persons with Disabilities, with expansion and greater integration of services and a more inclusive organization to overcome these challenges.

## INTRODUCTION

As one of the countries most affected by the covid-19 pandemic, Brazil accounts for the second highest number of deaths in the world^1^. Reflecting the profound social inequalities and implemented policies, the pandemic generated greater impacts on vulnerable populations, and can be characterized as a syndemic^2^.

Among the most vulnerable groups, people with disabilities (PWD) stand out, who tend to experience more precarious living conditions and face greater barriers to access public goods and services, including health. Such conditions make them more susceptible to the effects of the pandemic^3^.

Part of the PWD need continuous assistance at different care levels, with emphasis on rehabilitation, defined as “a set of measures that help people with disabilities or about to acquire disabilities to have and maintain an ideal functionality in the interaction with their environment”^4^. However, despite differences between countries, it is common for PWD health policies to face: lack of strategic planning, lack of resources and infrastructure; flaws in services management and in information production; barriers to access; difficulties in social participation of PWD and their families^4^.

This scenario has been aggravated by the pandemic in at least two ways. Initially, adapting protocols for the contingency of the disease, restricting the movement of people and redirecting resources to emergency areas affected the provision of elective services, such as rehabilitation and other care for PWD^5,6^, which increased waiting lists for care. On the other hand, there were more people in need of rehabilitation after prolonged hospitalizations and patients with post-covid-19 syndrome, or long-term covid, characterized by persistent symptoms from mild to severe presentation, after the acute phase of infection, resulting from the systemic involvement of the organism^7^. The close relationship of covid-19 with the production of disabilities leads to the need for a greater number of rehabilitation actions^8,9^.

Growing health care needs put even more pressure on overburdened health systems. In the Brazilian case, it is important to highlight that the first integrated and comprehensive care policy for people with disabilities, the Care Network for People with Disabilities (RCPD), dates from 2012 and still faces difficulties in its implementation. One of the central elements of this policy are the Specialized Rehabilitation Centers (CER), specialized care units that act as a regional reference in the health care network and were created as an innovation in the RCPD. CER are qualified to treat two or more types of disability (hearing, physical, intellectual and/or visual), in the multidisciplinary outpatient rehabilitation model^10^.

Considering the importance of the CER in the RCPD, the objectives of this article are to analyze the impact of the pandemic on the functioning of these services and provide subsidies to inform public policies aimed at guaranteeing PWD’s right to health.

## METHODS

This study analyzed the variation in the outpatient production of CER, recorded in the SIA/SUS between March 2019 and December 2021, and compared these results with CER managers’ perceptions about the impacts of the pandemic on their units.

A total of 247 procedures registered in the System of Management of the Table of Procedures, Medications and Ortheses/Prostheses and Special Materials (SIGTAP) of SUS, listed by the Rehabilitation Instruction of the Ministry of Health^11^ (2020) were selected for analysis. Data extraction was performed in March 2022 using the method developed by Saldanha *et al*.^12^. The procedures were organized into 18 groups: i) care for the person with a stoma; ii) group activity; iii) multidisciplinary assessment in visual rehabilitation; iv) medical consultation; v) consultation with a higher-level professional (except physicians); vi) Dispensation of Orthoses, Prostheses and Special Materials (OPM) for physical disabilities; vii) dispensation of OPM for visual disabilities; viii) dispensation of hearing aids; ix) provision of wheelchairs; x) diagnostic services; xi) cardiorespiratory physiotherapy; xii) supply of materials for ostomies; xiii) speech therapy; xiv) hearing therapy; xv) physical therapy; xvi) intellectual therapy; xvii) visual therapy; xviii) home visit. These procedures were also evaluated according to the performer’s professional category.

Data from all CER accredited by SUS in November 2019 were analyzed, according to information from the Department of Specialized and Thematic Care of the Ministry of Health Secretariat of Specialized Health Care, which presented production data in the selected period.

To assess the effect of the pandemic, monthly averages of the number of procedures were calculated in three periods: year before the pandemic in the country (YBP) from March 2019 to February 2020; first year of the pandemic (YP1) from March 2020 to February 2021; and second year of the pandemic (YP2) from March to December 2021.

An evolution in the number of procedures performed was observed by comparing the averages of YP1 and YBP, and between YP2 and YBP, using the following expression: $x=-\left(1-\frac{AP}{APP}\right)\cdot 100$ .

The results were contextualized and triangulated with the CER managers’ perception, measured by a web survey, applied between November 2020 and February 2021, on the Google Forms platform. To guarantee data quality, the research followed the criteria proposed by the Checklist for Reporting Results of Internet E-Surveys^13^.This is a non-probabilistic, convenience sample. Recruitment took place by telephone contact and email invitations to the CER accredited by SUS at the time. Individuals who identified themselves as CER “managers” were included. To prevent duplicate responses, the respondent’s e-mail address was used as the only marker.

The questionnaire prepared by the researchers contained 44 questions, of which 42 were closed, divided into the following themes: general characteristics of the respondent and the CER; functioning of the service during the pandemic; professional training; structure for telerehabilitation; care flow for post-covid-19 patients; social support. Open questions addressed the main challenges experienced and the strategies used for coping.

Only complete questionnaires were registered on the database, without weighting the questions. After consistency analysis, descriptive analysis of categorical variables was performed, presented in percentage results. For the open questions, there was identification of relevant thematic content and with greater repetition.

For questions about the impact of the pandemic on activities, the Likert scale was used. In this case, responses were converted into numerical data (1 = not affected, 2 = slightly affected, 3 = moderately affected and 4 = very affected). There was the answer option “did not perform” the activity previously, to filter the respondents who would be accounted for each type of activity. Results are presented as the average obtained from the set of valid responses. The analyses were conducted using the Rv3.5 software and followed these steps: i) content analysis applied to the answers to the questions, then submitted to pre-analysis, ii) exploration of the material and iii) treatment of the results, according to the main themes identified.

This study was carried out within the scope of the national research “Challenges of implementing the Care Network for Persons with Disabilities in different regional contexts: multidimensional and multiscale approach”, approved by the Ethics Committee of the Faculty of Public Health of the University of São Paulo, under the number 4,726,914.

## RESULTS

Table 1 presents the characteristics of the CER whose procedures were analyzed, as well as the CER that answered the web survey.

**Table 1:** Characteristics of the analyzed CER.

Variable	Analysis of SIA-SUS procedures	websurvey respondents
	
	n (%)	n (%)
Total CER	237 (100.0)	85 (100.0)
Brazilian region		
North	23 (9.7)	4 (4.7)
Northeast	79 (33.3)	24 (28.2)
Southeast	91 (38.4)	35 (41.2)
South	18 (7.6)	12 (14.1)
Midwest	26 (11.0)	10 (11.8)
Type		
II	153 (64.6)	55 (64.7)
III	57 (24.0)	19 (22.4)
IV	27 (11.4)	11 (12.9)
Type of disability served		
Hearing	103 (43.5)	40 (47.1)
Physical	217 (91.6)	77 (90.6)
Intellectual	204 (86.1)	71 (83.5)
Visual	58 (24.5)	24 (28.2)
Nature		
Public	125 (52.7)	36 (42.4)
Philanthropic	112 (47.3)	49 (57.6)

CER type II: services qualified to attend to two different types of disability; CER type III: services qualified to attend to three different types of disability; CER type IV: services qualified to meet the four types of disability.

Comparison between periods shows a decrease of 33.3% in the total number of procedures in the first year of the pandemic, that is, a loss of 178,700.33 monthly procedures (Table 2). After this period, there is a recovery and an increase of 10.7% in YP2.

**Table 2:** Average number of procedures performed in the CER by type. Brazil, 2019–2021.

	Monthly Average of Procedures			Difference between procedures performed (%)	
	
	YBP	YP1	YP2	YP1/YBP	YP2/YBP
Care for the person with a stoma	1,681.6	2,365.2	3,142.0	40.7	86.8
Supply of ostomy materials	11,898.1	13,071.1	14,927.8	9.9	25.5
Consultation of a higher-level professional (except physician)	58,305.3	60,121.8	93,163.6	3.1	59.8
Home visit	45.4	44.4	72.2	-2.2	59.0
Provision of wheelchairs	6,803.6	5,477.4	6,515.7	-19.5	-4.2
Multidisciplinary assessment	1,063.3	818.6	959.6	-23.0	-9.8
Medical appointment	41,872.7	31,531.4	46,384.3	-24.7	2.7
OPM dispensation - physical disability	6,734.3	5,094.2	7,309.9	-24.4	8.6
OPM dispensation - visual disability	833.1	549.5	805.2	-34.0	-3.3
Hearing aids dispensation	5,071.3	3,736.3	5,778.8	-26.3	13.9
Intellectual therapy	169,380.9	108,945.4	178,951.7	-35.7	5.7
Speech therapy	11,824.2	7,390.8	11,326.1	-37.5	-4.2
Diagnostic service	56,359.8	34,510.8	57,647.2	-38.8	2.3
Visual therapy	5,268.8	3,050.6	4,864.9	-42.1	-7.7
Hearing therapy	4,280.5	2,465.3	4,044.9	-42.4	-5.5
Physical therapy	151,381.5	77,836.3	153,161.4	-48.6	1.2
Cardiorespiratory physiotherapy	2,131.8	998.9	4,351.4	-53.1	104.1
Group activity	2246.3	474.1	1028.0	-78.9	-54.2
Total Procedures	537182.3	358482.0	594434.7	-33.3	10.7

YBP: year before the pandemic; YP1: first year of the pandemic; YP2: second year of the pandemic; OPM: orthoses, prostheses and special materials.

Source: SIA-SUS.

The greatest losses in YP1 occurred for collective and individual therapeutic care of all specialties, accompanied by diagnostic approaches and distribution of orthoses, prostheses and special materials (Table 2). Only care for people with a stoma and appointments by higher education professionals (non-physicians) did not suffer a reduction in the record of procedures in YP1, a trend maintained in YP2.

In the comparison between YP2 and YBP, recovery was observed in 11 of the 18 groups, with emphasis on the 104.1% increase in Cardiorespiratory Physiotherapy procedures. Group activities, multidisciplinary assessments, visual therapies, hearing therapies, speech therapy and provision of wheelchairs and OPM for visual disability had not yet recovered the average number of procedures in YP2.

The analysis of procedures by performing professional category reveals that only nursing procedures did not suffer a reduction in YP1. In YP2, dentists, pedagogues/psychopedagogues and physicians had not yet reached the average number of YBP procedures (Table 3).

**Table 3:** Procedures performed in the CER by professional category. Brazil, 2019–2021.

Variable	Monthly Average of Procedures			Difference between procedures performed (%)	
	
	YBP	YP1	YP2	YP1/YBP	YP2/YBP
Nurse	25,412.70	32,990.30	47,067.60	29.8	85.2
Physical educator	219.4	203.9	492.2	-7.1	124.3
Psychologist	47,830.10	36,632.50	60,074.70	-23.4	25.6
Social worker	26,514.90	19,658.00	30,964.80	-25.9	16.8
Nutritionist	4,244.20	3,042.40	5,346.70	-28.3	26
Speech-language-hearing therapist	100,431.00	69,098.70	110,952.20	-31.2	10.5
Occupational therapist	47,970.30	32,999.80	55,905.30	-31.2	16.5
Physician	89,267.60	57,848.30	88,111.00	-35.2	-1.3
Physiotherapist	169,527.30	91,526.30	170,593.10	-46	0.6
Pedagogue/Psychopedagogue	19,652.20	10,531.30	18,306.90	-46.4	-6.9
Dentist surgeon	1,285.10	542.6	948.2	-57.8	-26.2
Other professionals	2,389.50	1,807.20	3,542.30	-24.4	48.2
No identified CBO	2,379.70	1,588.00	2,077.20	-33.3	-12.7

YBP: year before the pandemic; YP1: first year of the pandemic; YP2: second year of the pandemic; CBO: Brazilian Classification of Occupations.

Source: SIA-SUS.

A total of 93 responses to the online form were received. Excluding repeated entries, a final number of 85 respondents was obtained, active in 34.3% of the total number of qualified CER; among them, physiotherapists (35.3%), speech-language-hearing therapists (12.9%), social workers (10.6%), psychologists (9.4%) administrators (7.1%) and other professionals (24.7%). Among the participants, 70.6% worked in CER with up to 50 professionals.

The perception of the responding managers is that all actions were affected, with greater damage to user group activities, visual therapies and home visits (Figure 1). When asked about the difficulties experienced, there were reports of services completely shut down in the initial moments of the pandemic.

**Figure:**
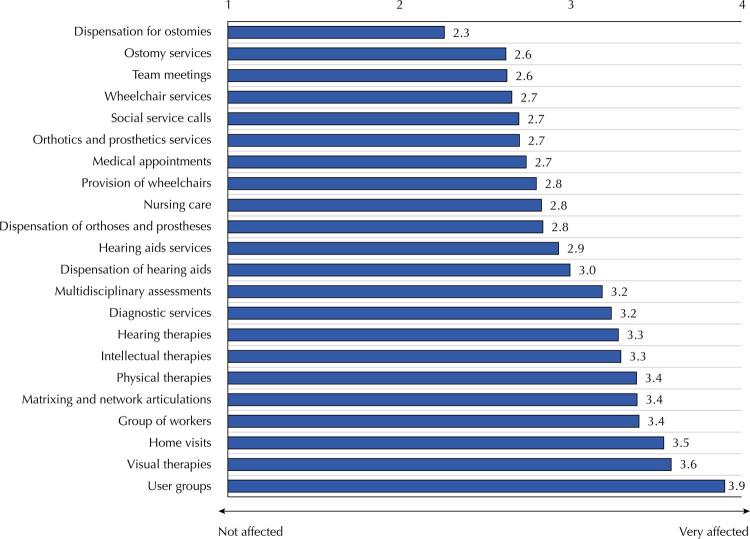
Managers’ perception of how much the activities carried out in the CER were affected by the Covid-19 pandemic. Brazil, 2021.

Regarding the organization of services during the pandemic, 91.7% of managers indicate structural and/or organizational changes in the units to adapt to the new reality (Table 4). In confirmation of the outpatient production data, a reduction in the number of services delivered (97.6%) was noted, as well as a reduction in the frequency of therapies for each patient (83.5%). In addition, adjustments were incorporated in the format of services and a decrease in the number of professionals who worked in their usual functions was observed.

**Table 4:** Modifications in the CER during the period of the covid-19 pandemic (N = 85).

Variable	Yes, n (%)	No, n (%)	Don’t know, n (%)
Unit operation			
Structural and/or organizational changes in the unit	78 (91.7)	7 (8.2)	0
Adaptations for priority and risk groups for covid-19	76 (89.4)	9 (10.6)	0
Reduction in the number of appointments made per day	83 (97.6)	2 (2.4)	0
Increased duration of each appointment	27 (31.8)	57 (67.0)	1 (1.2)
Increased interval between appointments	81 (95.3)	3 (3.5)	1 (1.2)
Reduction in the frequency of therapies for each patient	71 (83.5)	13 (15.3)	1 (1.2)
User complaints due to the queue to be attended to	33 (38.8)	49 (57.7)	3 (3.53)
Rehabilitation team			
Team rotation between shifts	49 (57.7)	36 (42.3)	0
Relocation of professionals to other units	35 (41.2)	50 (58.8)	0
Change of professionals’ attributions	51 (60.0)	34 (40.0)	0
Professional removal due to case or suspicion of covid-19	83 (97.6)	2 (2.4)	0
Psychological support for professionals	52 (61.1)	31 (36.5)	2 (2.4)
Telerehabilitation			
Conducting telehealth appointments	71 (83.5)	14 (16.5)	0
Sufficiency of computers with audio and video devices	41 (48.2)	44 (51.8)	0
Institutional cell phones and/or tablets	40 (47.1)	45 (52.9)	0
Adequate internet connection	58 (68.2)	27 (31.8)	0
Wireless network	54 (63.5)	31 (36.5)	0
Use of personal telephone by professionals	55 (64.7)	25 (29.4)	5 (5.9)

CER professionals were relocated to other units in 41.2% of cases. In 60.0% of the CER, workers started to perform other functions in the same unit. And there were removals of workers due to a condition or suspicion of covid-19 in 97.6% of the CER that answered the survey.

Criteria for prioritizing patients who should be seen in person were introduced. The most reported were: individuals at risk of injuries or delay in functionality; greater neuromotor impairment; people with dysphagia and; children in development stimulation. Acute conditions and care for people without comorbidities were also identified as priorities. For PWD with comorbidities, remote care was prioritized, with a view to reducing their exposure and the risk of contamination in the health service.

Most managers stated that there was professional training on: prevention and transmission of covid-19 (77.65%), use of PPE (84.71%) and care flow for covid-19 (63.53%).

At the moment when the web survey was conducted, half of the services were already providing care to people with covid-19 sequelae. In 81.4% of these services, such users were prioritized for scheduling, and in 55.8% a specific protocol was used. Care guidelines for post-covid-19 rehabilitation cases were received by 54.1% of managers. Structural adaptations in the unit to receive these cases were made in 34.1%. However, 63.5% stated that professional training on rehabilitation after covid-19 had not been offered.

Although 83.5% of the managers reported tele-assistance, technological resources for telerehabilitation were unavailable or insufficient in more than half of the units, which led professionals to use their cell phones for audio and video calls (68.7%). In 27.1% of cases, users did not have the structural conditions to be served virtually.

A survey of the served population’s health needs was carried out in 32.6% of the CER. Managers were not able to describe specific tools used in this diagnosis. Telephone contacts and remote consultations limited to the adaptation of rehabilitation procedures and occasional referrals to other services were reported. Only 9.4% mentioned articulation actions with the health network, territory and/or other sectors.

As part of the fight against the pandemic, 62.3% of the services carried out social assessment actions and directed users to social assistance resources (emergency income transfer, food and other programs). Deliberative council meetings for social participation were suspended or reduced in 42.9% of the units. Informational materials on the specificities of PWD and the covid-19 pandemic were produced and/or disseminated by 63.5% of the services.

In the open questions, there were reports of total shutdown of some services in the initial moments of the pandemic, in addition to struggles and ease regarding understanding and adhering to the new formats of care by users and their families. Insufficient adequate transport, fear of contamination and insecurity were some of the factors described by managers that would have led to the discontinuation of therapies, at the initiative of users. The teams’ performance in reinventing the ways of working and in the initiative to develop protocols for the new demands was highlighted.

Well-structured action plans and strategies, with systematized organization by the institution itself and integrated action with the health network actions to face the pandemic, were rarely described. Difficulties were reported to obtain instructions from higher authorities, lack of support for the introduction of telehealth services and registration and billing of this procedure, as well as pressure for the productivity of services that made it difficult to maintain biosafety protocols.

## DISCUSSION

Even before the covid-19 pandemic, difficulties in implementing the RCPD were already being discussed to guarantee equity in access and comprehensiveness of care^10,14^. The findings presented here demonstrate a profound decrease in the number of procedures performed, especially in the initial months of the pandemic, as well as a decrease in the offer of therapeutic groups and individual consultations, a high rate of professional leave and an insufficient structure for telerehabilitation. These can be configured as additional challenges for the consolidation of this network.

The results presented are in line with other studies in relation to impairments in accessing health services by PWD^6,15,16^. This impact can be even greater, since the “number of procedures” should not be confused with the “number of services” performed, because a single service can comprise more than one procedure.

There is a convergence between the managers’ perception and the losses verified for the outpatient production of CER in YP1. However, activities such as team meetings, networking and matrix support could not be compared due to the absence of codes for recording procedures at the time of data collection, even though they configure key actions in the formulation of strategies in a crisis situation. A similar situation occurs for multidisciplinary assessments in hearing, physical and intellectual rehabilitation. Such procedures are given to initial assessments of new users accessing rehabilitation services.

The procedure that underwent the greatest decrease during the analyzed period was that of “group activities”. The need for social distancing is a decisive factor for this finding. However, collective activities are essential for maintaining motor gains and preventing chronic diseases, in addition to providing socialization, exchange of experiences and knowledge. Prolonging non-performance of group activities may severely impact users’ physical, social and cognitive conditions.

Procedures aimed specifically at visual disability, as well as hearing and speech rehabilitation, have not returned to pre-pandemic performance levels, which denotes the risk of aggravating disparities in care for different types and levels of disability.

The increase in “Consultation of higher-level professionals (except doctors)” in YP2 can be explained by the wide use of this code, encompassing both face-to-face therapies and virtual ones implemented (especially in the initial absence of recognition of remote consultations, standardized only from the end of 2021). In addition, the code may have been used for individual calls originating from suspended groups.

Although in 2021 there was a greater wave of cases and deaths, there was an increase in some procedures to pre-YBP levels. This fact may be related to local guidelines for the resumption of elective outpatient care from the first half of 2021, concomitant with the start of vaccination and dissemination of prevention measures, especially the use of masks.

The high occurrence of professional leave due to covid-19 in the responding CER is similar to that observed in other countries^17^. The relocation of functions among workers, and from them to other health units, may also have made it difficult to maintain activities. In some municipalities, especially physiotherapists were displaced to cover the created hospital beds.

The sequelae of covid-19, due to their complexity and diversity, have presented themselves as an additional challenge for health care networks^18^. Patients hospitalized in intensive care units for long periods need to be included in rehabilitation programs to deal with the consequences of immobility and the use of mechanical ventilation^19^.

In Brazil, these cases of post-covid-19 functional disabilities started to be directed to rehabilitation services. This fact may explain the significant increase in physiotherapeutic procedures for respiratory and cardiovascular conditions, and procedures related to ostomy care, especially tracheostomies resulting from the need for prolonged intubation, corroborating Dinuzzi et al.^20^.

When responding to post-covid-19 demands, the CER undergo a change in the service profile. Cardiorespiratory rehabilitation was not an action commonly performed in the CER, since the service is oriented to attend to hearing, physical, intellectual and visual disabilities^11^. Even so, there was an effort to guarantee priority access to these patients, even if difficulties were identified for the reconfiguration of services to occur in a timely manner to adequately serve them.

This situation was not necessarily accompanied by the increase and training of teams or by the expansion of the capacity of services. Without network expansion, it is likely that other users will experience greater difficulties in ensuring the necessary care. The quality of care for new and old demands in rehabilitation will depend on the instrumentalization of multidisciplinary teams, through permanent education, evaluation and monitoring of changes in work processes, and the matrix of cases and experiences.

Although telerehabilitation is identified as a promising strategy for maintaining assistance to PWD during the pandemic^21^, the results show that part of the services did not have adequate and institutionally guaranteed conditions for the use of this tool.

The use of telehealth in Brazil is challenging due to regulatory and structural factors^22^. Teleconsultations were authorized by class councils and the Ministry of Health based on the tensions generated by the pandemic. Also, new work tools had to be quickly assimilated by health professionals and services.

Although most respondents state that users are able to access telerehabilitation, it is necessary to highlight that the CER are concentrated in state capitals and municipalities with better infrastructure^10^. With precarious internet access in many Brazilian locations, telerehabilitation programs could hardly be applied extensively, and would run the risk of discriminating against the digitally excluded population if there are no significant changes in public policies^23^.

Protocols and guidelines for the general population were replicated without customization for PWD and their reference services^24^. The specificities within this heterogeneous group were not covered by the coping plans, including regarding even greater vulnerabilities, such as institutionalized PWD, living on the streets, immigrants and women^25^. Such negligence reflects a process of systematic invisibility^26^. Despite the recommendations for the adoption of inclusive measures, based on the logic of the law^3,27^, no data were observed to suggest this implementation.

Most respondents stated that they carried out some type of action aimed at providing social support to their users during isolation, which can be considered positive as compared to the fragility of social support actions in coping with covid-19 identified in other areas of assistance^28^.

The analysis of public policies adopted during the pandemic period, as well as future reformulations, depends on the production of reliable and specific epidemiological information on the population living with disabilities. Failure to meet this assumption can configure, in itself, a mechanism of social exclusion, since disaggregated data on the involvement of covid-19 by status, type and severity of disability are rare, although extremely necessary^29^.

One of the limits of this study is inherent to the use of SIA-SUS data. Even though there may be a delay in recording procedures, it is an information system with considerable agility that should be used for planning, supply analysis, coverage and selection of priorities^30^.

Another known limitation is that the web survey did not use a probabilistic sample. Even so, CER responses were obtained with characteristics similar to those of the services available in the country (Table 1). It is believed that the results bring important elements and contextualize the production data. The existing regional differences in the country can and should be objects of new studies, aiming to identify the local specificities.

By overlapping two sources of information, secondary data and managers’ perceptions, the results obtained by analyzing the former come to life, bringing them closer to reality with the use of a methodological approach pertinent to the proposed objective. Understanding the reality of PWD care in the country, including territories without CER, and variations within the different stages of the pandemic, requires targeted studies.

## CONCLUSION

In the early months of the covid-19 pandemic, there was a severe impact on the ability to provide services in the CER in Brazil. Such effects may have also occurred on other rehabilitation services. In addition to the reduction in rehabilitation procedures and consultations, substantial changes were observed in the functioning of the teams, which led to the damming of existing demands.

The results show a scenario that needs to be changed and point out ways in which policy makers and service managers can follow to improve care for PWD.

Rehabilitation services will have to deal not only with the inclusion of new treatment protocols for the post-covid-19 patient, but also with attending to: users who have suffered consequences from the decrease in volume, or forced interruption, of their therapies; those who present losses resulting from health conditions aggravated by isolation; in addition to all those referred by new diagnoses of congenital and acquired deficiencies. In short, the scenario is one of extreme overload for these services.

The covid-19 pandemic highlights the need to strengthen the SUS and the RCPD, through expansion and integration of health services, with co-responsibility between primary health care and specialized, hospital and rehabilitation care. Favoring the full exercise of autonomy and functional capacity can only be achieved through broad social protection and inclusive social organization, during and after the pandemic.
