# Supplementary material for: Specialized Rehabilitation Centers (CER) in the SUS and the impact of the covid-19 pandemic
Source: Rev Saude Publica. 2023 May 11;57(Suppl 1):9s. doi: 10.11606/s1518-8787.2023057004807 (PMC10185313; doi:10.11606/s1518-8787.2023057004807)
Supplement: Translation

# Translation: Centros Especializados em Reabilitação (CER) no SUS e o impacto da pandemia de covid-19

**Authors:** Bianca Tomi Rocha Suda, Paulo Henrique dos Santos Mota, Aylene Bousquat

**Affiliations:** I Brasil Universidade de São Paulo. Faculdade de Saúde Pública. Programa de Pós-Graduação em Saúde Pública. São Paulo, SP, Brasil; II Brasil Universidade de São Paulo. Faculdade de Saúde Pública. Departamento de Política, Gestão e Saúde. São Paulo, SP, Brasil

**Keywords:** Centros de Reabilitação, Sistema Único de Saúde, Acesso aos Serviços de Saúde, COVID-19

## Abstract

**OBJETIVO:**

Analisar o impacto da pandemia de covid-19 no funcionamento dos Centros Especializados em Reabilitação (CER) no SUS.

**MÉTODOS:**

Realizou-se análise da variação da produção ambulatorial dos CER com base nos dados do Sistema de Informações Ambulatoriais do Sistema Único de Saúde (SIA-SUS) no período de março de 2019 a dezembro de 2021. Tais resultados foram cotejados com as percepções de gestores de CER acerca dos impactos da pandemia nas unidades, aferidas por *websurvey* aplicado entre novembro de 2020 e fevereiro de 2021. Foram calculadas as médias mensais de 247 procedimentos, organizados em 18 grupos, para três períodos – ano anterior à pandemia (APP), primeiro (AP1) e segundo (AP2) anos de pandemia. Por meio do formulário on-line foram coletadas informações sobre: funcionamento e organização dos serviços; reabilitação pós-covid-19; ações de apoio às necessidades de usuários e profissionais; estratégias e desafios vivenciados.

**RESULTADOS:**

Houve redução de 33,3% do total de procedimentos em AP1 em relação a APP. Não foram reduzidos procedimentos realizados por enfermeiros e para pessoas ostomizadas. Ocorreu maior prejuízo para atividades em grupo, terapias visuais e visitas domiciliares. Em AP2, houve recuperação das médias em relação a APP em 11 grupos de procedimentos, com aumento de 104,1% de Fisioterapia Cardiorrespiratória. Nas respostas ao formulário on-line, 91,7% dos gestores indicam mudanças estruturais e/ou organizacionais nos CER como: criação de critérios de priorização para atendimentos; introdução de telerreabilitação; modificações no processo de trabalho e; disponibilização de capacitações profissionais. Metade dos CER já atendiam pessoas com sequelas de covid-19, mas nem todos haviam sido instrumentalizados para tanto. Identificaram-se limitações na participação e no suporte social para PCD.

**CONCLUSÕES:**

Houve severo impacto da pandemia de covid-19 nos CER. Ao represamento de demandas prévias se somam aquelas de usuários pós-covid-19, configurando um quadro desafiador. Faz-se necessário fortalecimento da Rede de Cuidados à Pessoa com Deficiência, com ampliação e maior integração dos serviços e organização mais inclusiva para a superação desses desafios.

## INTRODUÇÃO

O Brasil é um dos países mais afetados pela pandemia de covid-19, e responde pelo segundo maior número de mortes no mundo^1^. Reflexo das profundas desigualdades sociais e das políticas implementadas, a pandemia gerou impactos maiores sobre populações vulneráveis, e pode ser caracterizada como uma sindemia^2^.

Dentre os grupos mais vulneráveis, o das pessoas com deficiência (PCD) se sobressai, visto que tende a experimentar condições de vida mais precárias e enfrentar maiores barreiras de acesso a bens e serviços públicos, inclusive de saúde. Tais condicionantes as tornam mais suscetíveis aos efeitos da pandemia^3^.

Parcela das PCD necessita de cuidados continuados em diferentes níveis assistenciais, com destaque para reabilitação, definida como “um conjunto de medidas que ajudam pessoas com deficiência ou prestes a adquirir deficiências a terem e manterem uma funcionalidade ideal na interação com seu ambiente”^4^. Contudo, apesar de diferenças entre países, é comum que as políticas de saúde para PCD enfrentem: ausência de planejamento estratégico, falta de recursos e infraestrutura; falhas na gestão de serviços e na produção de informações; barreiras ao acesso; baixo envolvimento das PCD e seus familiares^4^.

Este cenário foi agravado pela pandemia em pelo menos duas direções. Inicialmente, a adequação aos protocolos para contingência da doença, restrição da circulação de pessoas e redirecionamento de recursos para áreas emergenciais afetaram a oferta de serviços eletivos, como a reabilitação e outros cuidados para PCD^5,6^, o que aumentou filas de espera por atendimento. Por outro lado, houve maior número de pessoas que necessitam de reabilitação após internações prolongadas e de pacientes com síndrome pós-covid-19, ou covid longa, caracterizada por sintomas persistentes de apresentação leve a severa, após a fase aguda de infecção, decorrentes do acometimento sistêmico do organismo^7^. A estreita relação da covid-19 com a produção de deficiências leva à necessidade de um maior número de ações de reabilitação^8,9^.

O aumento das necessidades de saúde pressiona ainda mais os sobrecarregados sistemas de saúde. No caso brasileiro, é relevante destacar que a primeira política integrada e abrangente de cuidados às PCD, a Rede de Cuidados à Pessoa com Deficiência (RCPD), data de 2012 e ainda enfrenta dificuldades em sua implementação. Um dos elementos centrais desta política são os Centros Especializados em Reabilitação (CER), unidades de atenção especializada que atuam como referência regional da rede de atenção à saúde e foram criadas como inovação na RCPD. Os CER são habilitados a atender dois ou mais tipos de deficiência (auditiva, física, intelectual e/ou visual), no modelo de reabilitação ambulatorial multiprofissional^10^.

Considerando a importância dos CER na RCPD, os objetivos do presente artigo são analisar o impacto da pandemia no funcionamento desses serviços e fornecer subsídios para informar políticas públicas voltadas à garantia do direito à saúde das PCD.

## MÉTODOS

Este estudo analisou a variação da produção ambulatorial dos CER, registrada no SIA/SUS entre março de 2019 e dezembro de 2021, e cotejou tais resultados com as percepções de gestores de CER acerca dos impactos da pandemia em suas unidades.

Foram selecionados para análise 247 procedimentos registrados no Sistema de Gerenciamento da Tabela de Procedimentos, Medicamentos e Órteses/Próteses e Materiais Especiais (SIGTAP) do SUS, elencados pelo Instrutivo de Reabilitação do Ministério da Saúde^11^ (2020). A extração dos dados foi realizada em março de 2022 pelo método elaborado por Saldanha *et al*.^12^. Os procedimentos foram organizados em 18 grupos: i) atendimento à pessoa ostomizada; ii) atividade em grupo; iii) avaliação multiprofissional em reabilitação visual; iv) consulta médica; v) consulta de profissional de nível superior (exceto médico); vi) dispensação de Órteses, Próteses e Materiais Especiais(OPM) para deficiência física; vii) dispensação de OPM para deficiência visual; viii) dispensação de aparelhos auditivos; ix) dispensação de cadeiras de rodas; x) exame diagnóstico; xi) fisioterapia cardiorrespiratória; xii) fornecimento de materiais para ostomias; xiii) terapia fonoaudiológica; xiv) terapia-reabilitação auditiva; xv) terapia-reabilitação física; xvi) terapia-reabilitação intelectual; xvii) terapia-reabilitação visual; xviii) visita domiciliar. Esses procedimentos foram ainda avaliados segundo a categoria profissional do executante.

Foram analisados dados de todos os CER habilitados em novembro de 2019, segundo informações do Departamento de Atenção Especializada e Temática da Secretaria de Atenção Especializada em Saúde do Ministério da Saúde, que apresentaram dados de produção no período selecionado.

Para avaliar o efeito da pandemia, foram calculadas médias mensais do número de procedimentos em três períodos: ano anterior à pandemia no país (AAP) de março de 2019 a fevereiro de 2020; primeiro ano de pandemia (AP1) de março de 2020 a fevereiro de 2021; e segundo ano de pandemia (AP2) de março a dezembro de 2021.

Observou-se a evolução do número de procedimentos realizados pela comparação entre médias de AP1 e APP, e entre AP2 e APP, utilizando-se a seguinte expressão: $x=-\left(1-\frac{AP}{APP}\right)\cdot 100$ .

Os resultados foram contextualizados e triangulados com a percepção de gestores dos CER, aferida por formulário on-line, aplicado entre novembro de 2020 e fevereiro de 2021, na plataforma Google Forms. Para garantia da qualidade dos dados, a pesquisa seguiu os critérios propostos pelo *Checklist for Reporting Results of Internet E-Surveys*^13^.

Trata-se de uma amostra não probabilística, de conveniência. O recrutamento se deu por contato telefônico e convites por e-mail para os CER habilitados à época. Foram incluídos indivíduos que se identificaram como “gestores” de CER. Para prevenir respostas duplicadas, utilizou-se o endereço de e-mail do respondente como marcador único.

O questionário elaborado pelos pesquisadores continha 44 perguntas, das quais 42 eram fechadas, divididas entre os seguintes eixos temáticos: características gerais do respondente e do CER; funcionamento do serviço durante a pandemia; capacitação profissional; estrutura para telerreabilitação; fluxo assistencial para paciente pós-covid-19; apoio social. Questões abertas abordavam os principais desafios vivenciados e as estratégias utilizadas para o enfrentamento.

Somente questionários completos foram registrados no banco de dados, sem ponderação de valor às questões. Após análise de consistência, realizou-se análise descritiva das variáveis categóricas, apresentada em resultados percentuais. Para as perguntas abertas, houve identificação dos conteúdos temáticos relevantes e com maior repetição.

Nas questões sobre o impacto da pandemia nas atividades foi empregada a escala de Likert. Neste caso, as respostas foram convertidas em dados numéricos (1 = não afetadas, 2 = pouco afetadas, 3 = moderadamente afetadas e 4 = muito afetadas). Havia a opção de resposta “não realizava” a atividade previamente, para filtrar os respondentes que seriam contabilizados em cada tipo de atividade. Os resultados são apresentados pela média obtida do conjunto de respostas válidas. As análises foram conduzidas usando o *software* Rv3.5 e seguiram estas etapas: i) análise de conteúdo aplicada às respostas das questões, depois submetidas a pré-análise, ii) exploração do material e iii) tratamento dos resultados, segundo as principais temáticas identificadas.

Este estudo foi executado no âmbito da pesquisa nacional “Desafios da implementação da Rede de Cuidados à Pessoa com Deficiência em diferentes contextos regionais: abordagem multidimensional e multiescalar”, aprovada pelo Comitê de Ética da Faculdade de Saúde Pública da Universidade de São Paulo, sob o número 4.726.914.

## RESULTADOS

Na Tabela 1 são apresentadas as características dos CER cujos procedimentos foram analisados, assim como dos CER respondentes do *websurvey.*

**Tabela 1:** Características dos CER analisados.

Variável	Análise de procedimentos SIA-SUS	Respondentes do websurvey
	
	n (%)	n (%)
Total CER	237 (100,0)	85 (100,0)
Região brasileira		
Norte	23 (9,7)	4 (4,7)
Nordeste	79 (33,3)	24 (28,2)
Sudeste	91 (38,4)	35 (41,2)
Sul	18 (7,6)	12 (14,1)
Centro-Oeste	26 (11,0)	10 (11,8)
Tipo		
II	153 (64,6)	55 (64,7)
III	57 (24,0)	19 (22,4)
IV	27 (11,4)	11 (12,9)
Modalidade de deficiência atendida		
Auditiva	103 (43,5)	40 (47,1)
Física	217 (91,6)	77 (90,6)
Intelectual	204 (86,1)	71 (83,5)
Visual	58 (24,5)	24 (28,2)
Natureza		
Pública	125 (52,7)	36 (42,4)
Filantrópica	112 (47,3)	49 (57,6)

CER tipo II: serviços habilitados a atender duas diferentes modalidades de deficiência; CER tipo III: serviços habilitados a atender três diferentes modalidades de deficiência; CER tipo IV: serviços habilitados a atender as quatro modalidades de deficiência.

A comparação entre períodos evidencia diminuição de 33,3% no número total de procedimentos no primeiro ano de pandemia, ou seja, perda de 178.700,33 procedimentos mensais (Tabela 2). Após esse período, observa-se recuperação e acréscimo de 10,7% em AP2.

**Tabela 2:** Número médio de procedimentos realizados nos CER por tipo. Brasil, 2019–2021.

	Média Mensal de Procedimentos			Diferença entre procedimentos realizados (%)	
	
	AAP	AP1	AP2	AP1/APP	AP2/APP
Atendimento à pessoa ostomizada	1.681,6	2.365,2	3.142,0	40,7	86,8
Fornecimento de materiais para ostomias	11.898,1	13.071,1	14.927,8	9,9	25,5
Consulta de profissional nível superior (exceto médico)	58.305,3	60.121,8	93.163,6	3,1	59,8
Visita domiciliar	45,4	44,4	72,2	-2,2	59,0
Dispensação de cadeiras de rodas	6.803,6	5.477,4	6.515,7	-19,5	-4,2
Avaliação multiprofissionais	1.063,3	818,6	959,6	-23,0	-9,8
Consulta médica	41.872,7	31.531,4	46.384,3	-24,7	2,7
Dispensação de OPM deficiência física	6.734,3	5.094,2	7.309,9	-24,4	8,6
Dispensação de OPM deficiência visual	833,1	549,5	805,2	-34,0	-3,3
Dispensação de aparelhos auditivos	5.071,3	3.736,3	5.778,8	-26,3	13,9
Terapia em reabilitação intelectual	169.380,9	108.945,4	178.951,7	-35,7	5,7
Terapia fonoaudiológica	11.824,2	7.390,8	11.326,1	-37,5	-4,2
Exame diagnóstico	56.359,8	34.510,8	57.647,2	-38,8	2,3
Terapia em reabilitação visual	5.268,8	3.050,6	4.864,9	-42,1	-7,7
Terapia em reabilitação auditiva	4.280,5	2.465,3	4.044,9	-42,4	-5,5
Terapia em reabilitação física	151.381,5	77.836,3	153.161,4	-48,6	1,2
Fisioterapia cardiorrespiratória	2.131,8	998,9	4.351,4	-53,1	104,1
Atividade em grupo	2246,3	474,1	1028,0	-78,9	-54,2
Total Procedimentos	537182,3	358482,0	594434,7	-33,3	10,7

AAP: ano anterior Pandemia; AP1: primeiro ano de Pandemia; AP2: segundo ano de Pandemia; OPM: órteses, próteses e materiais especiais.

Fonte: SIA-SUS.

As maiores perdas em AP1 ocorreram para atendimentos terapêuticos coletivos e individuais de todas as especialidades, acompanhados por abordagens diagnósticas e dispensação de órteses, próteses e materiais especiais (Tabela 2). Apenas os cuidados às pessoas ostomizadas e consultas de profissionais de nível superior (não médicos) não sofreram redução no registro de procedimentos em AP1, tendência mantida em AP2.

Na comparação entre AP2 e AAP, observa-se recuperação de 11 dos 18 grupos, com destaque para o aumento de 104,1% dos procedimentos de Fisioterapia Cardiorrespiratória. As atividades em grupo, avaliações multiprofissionais, terapias visuais, terapias auditivas, terapia fonoaudiológica e dispensação de cadeiras de rodas e de OPM para deficiência visual ainda não haviam recuperado o número médio de procedimentos em AP2.

A análise dos procedimentos por categoria profissional executante revela que apenas os procedimentos de enfermagem não sofreram redução em AP1. Em AP2, cirurgiões dentistas, pedagogos/psicopedagogos e médicos ainda não haviam alcançado a média de procedimentos de APP (Tabela 3).

**Tabela 3:** Procedimentos realizados nos CER por categoria profissional. Brasil, 2019–2021.

Variável	Média Mensal de Procedimentos			Diferença entre procedimentos realizados (%)	
	
	AAP	AP1	AP2	AP1/APP	AP2/APP
Enfermeiro(a)	25.412,70	32.990,30	47.067,60	29,8	85,2
Educador(a) físico(a)	219,4	203,9	492,2	-7,1	124,3
Psicólogo(a)	47.830,10	36.632,50	60.074,70	-23,4	25,6
Assistente social	26.514,90	19.658,00	30.964,80	-25,9	16,8
Nutricionista	4.244,20	3.042,40	5.346,70	-28,3	26
Fonoaudiólogo(a)	100.431,00	69.098,70	110.952,20	-31,2	10,5
Terapeuta ocupacional	47.970,30	32.999,80	55.905,30	-31,2	16,5
Médico(a)	89.267,60	57.848,30	88.111,00	-35,2	-1,3
Fisioterapeuta	169.527,30	91.526,30	170.593,10	-46	0,6
Pedagogo(a)/ Psicopedagogo(a)	19.652,20	10.531,30	18.306,90	-46,4	-6,9
Cirurgião(ã) dentista	1.285,10	542,6	948,2	-57,8	-26,2
Outros profissionais	2.389,50	1.807,20	3.542,30	-24,4	48,2
Sem CBO identificado	2.379,70	1.588,00	2.077,20	-33,3	-12,7

AAP: ano anterior à pandemia; AP1: primeiro ano de pandemia; AP2: segundo ano de pandemia; CBO: Classificação Brasileira de Ocupações.

Fonte: SIA-SUS.

Foram recebidas 93 respostas ao formulário on-line. Excluídas as entradas repetidas, obteve-se o número final de 85 respondentes, atuantes em 34,3% do total de CER habilitados; dentre eles, fisioterapeutas (35,3%), fonoaudiólogos (12,9%), assistentes sociais (10,6%), psicólogos (9,4%) administradores (7,1%) e outros profissionais (24,7%). Entre os participantes, 70,6% atuavam em CER com até 50 profissionais.

A percepção dos gestores respondentes é de que todas as ações foram afetadas, com maior prejuízo para as atividades em grupo de usuários, terapias visuais e visitas domiciliares. Quando questionados sobre as dificuldades vivenciadas, houve relatos de serviços totalmente paralisados nos momentos iniciais da pandemia.

Em relação à organização dos serviços durante a pandemia, 91,7% dos gestores indicam mudanças estruturais e/ou organizacionais nas unidades para adaptação à nova realidade (Tabela 4). Em confirmação aos dados de produção ambulatorial, foi apontada redução nos atendimentos prestados (97,6%) assim como redução na frequência de terapias para cada paciente (83,5%). Ademais, foram incorporadas adequações no formato de atendimentos e observou-se diminuição no número de profissionais que atuavam em suas funções habituais.

**Tabela 4:** Modificações nos CER durante o período da pandemia de covid-19 (N = 85).

Variável	Sim, n (%)	Não, n (%)	Não sabe, n (%)
Funcionamento da unidade			
Mudanças estruturais e/ou organizacionais na unidade	78 (91,7)	7 (8,2)	0
Adaptações para grupos prioritários e de risco para covid-19	76 (89,4)	9 (10,6)	0
Redução do nº de atendimentos realizados por dia	83 (97,6)	2 (2,4)	0
Aumento da duração de cada atendimento	27 (31,8)	57 (67,0)	1 (1,2)
Aumento do intervalo entre atendimentos	81 (95,3)	3 (3,5)	1 (1,2)
Redução na frequência de terapias de cada paciente	71 (83,5)	13 (15,3)	1 (1,2)
Reclamações de usuários devido à fila de espera por atendimento	33 (38,8)	49 (57,7)	3 (3,53)
Equipe de reabilitação			
Revezamento entre turnos das equipes	49 (57,7)	36 (42,3)	0
Remanejamento de profissionais para outras unidades	35 (41,2)	50 (58,8)	0
Mudança de atribuições dos profissionais	51 (60,0)	34 (40,0)	0
Afastamento profissional por caso ou suspeita de covid-19	83 (97,6)	2 (2,4)	0
Suporte psicológico para os profissionais	52 (61,1)	31 (36,5)	2 (2,4)
Telerreabilitação			
Realização de teleatendimentos	71 (83,5)	14 (16,5)	0
Suficiência de computadores com dispositivos de áudio e vídeo	41 (48,2)	44 (51,8)	0
Suficiência de celulares e/ou *tablets* institucionais	40 (47,1)	45 (52,9)	0
Adequada conexão à internet	58 (68,2)	27 (31,8)	0
Rede *wireless*	54 (63,5)	31 (36,5)	0
Uso de telefone pessoal pelos profissionais	55 (64,7)	25 (29,4)	5 (5,9)

Houve realocação de profissionais dos CER para outras unidades em 41,2% dos casos. Em 60,0% dos CER, trabalhadores passaram a desempenhar outras funções na mesma unidade. E houve afastamento de trabalhadores devido a quadro ou suspeita de covid-19 em 97,6% dos CER que responderam à pesquisa.

Critérios para priorização dos pacientes que deveriam ser atendidos presencialmente foram introduzidos. Os mais relatados foram: indivíduos com risco de agravos ou de atraso na funcionalidade; maior comprometimento neuromotor; disfágicos e; crianças em estimulação do desenvolvimento. Quadros agudos e o atendimento de pessoas sem comorbidades também foram apontados como prioridades. Para as PCD com comorbidades foram priorizados atendimentos à distância, com vistas a diminuir sua exposição e o risco de contaminação no serviço de saúde.

**Figura:** Percepção dos gestores sobre quanto as atividades realizadas nos CER foram afetadas pela pandemia de Covid-19. Brasil, 2021.

A maior parte dos gestores afirmou que houve capacitação profissional sobre: prevenção e transmissão da covid-19 (77,65%), uso de EPI (84,71%) e fluxo assistencial para covid-19 (63,53%).

No momento de aplicação do *websurvey*, metade dos serviços já realizava atendimentos de pessoas com sequelas de covid-19. Em 81,4% destes serviços tais usuários eram priorizados para agendamento, e em 55,8% protocolo específico era empregado. As diretrizes assistenciais para casos de reabilitação pós-covid-19 foram recebidas por 54,1% dos gestores. Adaptações estruturais na unidade para receber estes casos foram feitas em 34,1%. No entanto, 63,5% afirmaram que não havia sido ofertada capacitação profissional sobre reabilitação após covid-19.

Embora 83,5% dos gestores relatem a realização de teleatendimentos, recursos tecnológicos para telerreabilitação eram indisponíveis ou insuficientes em mais da metade das unidades, o que levou profissionais a utilizar os próprios celulares para chamadas de áudio e vídeo (68,7%). Em 27,1% dos casos, os usuários não tinham condições estruturais para serem atendidos virtualmente.

O levantamento das necessidades em saúde da população atendida foi feito em 32,6% dos CER. Gestores não foram capazes de descrever ferramentas específicas utilizadas nesse diagnóstico. Relataram-se contatos telefônicos e atendimentos remotos limitados à adequação de condutas de reabilitação e eventuais encaminhamentos para outros serviços. Apenas 9,4% mencionaram ações de articulação com a rede de saúde, território e/ou outros setores.

Como parte do enfrentamento da pandemia, 62,3% dos serviços realizaram ações de avaliação social e direcionamento dos usuários para recursos da assistência social (programas emergenciais de transferência de renda, alimentação e outros). Reuniões de conselhos deliberativos para participação social foram suspensas ou reduzidas em 42,9% das unidades. Foram produzidos e/ou divulgados materiais informativos sobre especificidades das PCD e a pandemia de covid-19 por 63,5% dos serviços.

Nas questões abertas, houve relatos de paralisação total de alguns serviços nos momentos iniciais da pandemia, além de dificuldades e facilidades quanto à compreensão e aderência aos novos formatos de atendimentos pelos usuários e suas famílias. A insuficiência de transporte adequado, o medo de contaminação e insegurança foram alguns fatores descritos pelos gestores que teriam levado à descontinuidade das terapias, por iniciativa dos usuários. Foi destacada a atuação das equipes na reinvenção dos modos de atuar e na iniciativa de desenvolver protocolos para as novas demandas.

Raramente foram descritos planos de ação e estratégias bem estruturadas, com organização sistematizada da própria instituição e atuação integrada às ações da rede de saúde para o enfrentamento à pandemia. Foram reportadas dificuldades para obter instruções por instâncias superiores, falta de suporte para introdução do teleatendimento e para registro e faturamento desse procedimento, bem como pressão pela produtividade dos serviços que dificultou a manutenção dos protocolos de biossegurança.

## DISCUSSÃO

Antes mesmo da pandemia de covid-19 já se discutiam dificuldades de implantação da RCPD para garantir equidade no acesso e integralidade do cuidado^10,14^. Os achados aqui apresentados demonstram uma profunda diminuição dos procedimentos realizados, sobretudo nos meses iniciais da pandemia, bem como a diminuição da oferta de grupos terapêuticos e atendimentos individuais, alto índice de afastamento de profissionais e estrutura insuficiente para telerreabilitação. Estes podem se configurar como desafios adicionais para a consolidação dessa rede.

Os resultados apresentados vão ao encontro de outros estudos em relação aos prejuízos no acesso a serviços de saúde pelas PCD^6,15,16^. Esse impacto pode ser ainda maior, uma vez que o “número de procedimentos” não deve ser confundido com o “número de atendimentos” executados, já que um único atendimento pode compreender mais de um procedimento.

Nota-se convergência entre a percepção dos gestores e as perdas verificadas para a produção ambulatorial dos CER em AP1. Contudo, atividades como reuniões de equipe, articulação em rede e matriciamento não puderam ser comparadas pela ausência de códigos para registros dos procedimentos na época do levantamento dos dados, mesmo que configurem ações-chave na formulação de estratégias em situação de crise. Situação semelhante ocorre para avaliações multiprofissionais em reabilitação auditiva, física e intelectual. Tais procedimentos são dados a avaliações iniciais de novos usuários acessando os serviços de reabilitação.

O procedimento que sofreu maior diminuição, durante o período analisado, foi o de “atividades em grupo”. A necessidade de distanciamento social é fator decisivo para tal achado. No entanto, as atividades coletivas são essenciais para manutenção dos ganhos motores e para prevenção dos agravos crônicos, além de propiciar socialização, trocas de experiências e de saberes. O prolongamento da não realização das atividades em grupo poderá impactar severamente as condições físicas, sociais e cognitivas dos usuários.

Procedimentos voltados especificamente para deficiência visual, assim como reabilitação auditiva e terapia fonoaudiológica, não retomaram patamares de execução anteriores à pandemia, o que denota o risco de agravar disparidades na assistência para diferentes tipos e níveis de deficiência.

O incremento de “Consulta de profissionais de nível superior (exceto médico)” em AP2 pode ser explicado pela ampla utilização deste código, englobando tanto as terapias presenciais mantidas quanto as virtuais implantadas (especialmente na ausência inicial de reconhecimento dos atendimentos remotos, normatizados somente a partir do final do 2021). Além disso, o código pode ter sido utilizado para atendimentos individuais originados a partir de grupos suspensos.

Ainda que no ano de 2021 tenha ocorrido uma maior onda de casos e mortes, houve aumento de alguns procedimentos a níveis anteriores a APP. Este fato pode estar relacionado às orientações locais de retomada de atendimentos ambulatoriais eletivos a partir do primeiro semestre de 2021, concomitantes ao início da vacinação e divulgação de medidas de prevenção, sobretudo o uso de máscaras.

A elevada ocorrência de afastamento profissional por covid-19 nos CER respondentes é similar à verificada em outros países^17^. O remanejamento de funções entre os trabalhadores, e destes para outras unidades de saúde também pode ter dificultado a manutenção das atividades. Em alguns municípios, especialmente os fisioterapeutas foram deslocados para cobrir os leitos hospitalares criados.

As sequelas de covid-19, por sua complexidade e diversidade, têm se apresentado como um desafio adicional para as redes de atenção à saúde^18^. Pacientes hospitalizados em unidades de terapia intensiva por longos períodos necessitam ser incluídos em programas de reabilitação para lidar com as consequências do imobilismo e do uso de ventilação mecânica^19^.

No Brasil, estes casos de incapacidades funcionais pós-covid-19 passaram a ser direcionados para serviços de reabilitação. Este fato pode explicar o expressivo aumento dos procedimentos fisioterapêuticos para quadros respiratórios e cardiovasculares, e dos procedimentos relacionados aos cuidados de ostomias, sobretudo as traqueostomias resultantes da necessidade de intubação prolongada, corroborando Dinuzzi *et al* ^20^.

Ao responder pelas demandas pós-covid-19, os CER passam por mudança no perfil de atendimento. A reabilitação cardiorrespiratória não era uma ação comumente realizada nos CER já que o serviço é orientado para o atendimento das deficiências auditiva, física, intelectual e visual^11^. Ainda assim, houve esforço de garantir o acesso prioritário a estes pacientes, mesmo que se identifiquem dificuldades para que a reconfiguração dos serviços ocorresse em tempo hábil para atendê-los adequadamente.

Tal situação não necessariamente foi acompanhada pelo aumento e capacitação das equipes ou pela expansão da capacidade de atendimento dos serviços. Sem expansão da rede, é provável que outros usuários vivenciem maiores dificuldades para garantir o cuidado necessário. A qualidade do atendimento para novas e antigas demandas em reabilitação dependerá da instrumentalização das equipes multiprofissionais, por meio de educação permanente, avaliação e acompanhamento das modificações nos processos de trabalho, e do matriciamento de casos e experiências.

Embora a telerreabilitação seja identificada como estratégia promissora de manutenção da assistência às PCD durante a pandemia^21^, os resultados demonstram que parte dos serviços não apresentava condições adequadas, e institucionalmente garantidas, para a utilização dessa ferramenta.

O uso da telessaúde no Brasil é desafiador devido a fatores regulatórios e estruturais^22^. As teleconsultas foram autorizadas por conselhos de classe e pelo Ministério da Saúde a partir dos tensionamentos gerados pela pandemia. Ainda, novos instrumentos de trabalho tiveram de ser rapidamente assimilados por profissionais e serviços de saúde.

Apesar de a maioria dos respondentes afirmar que os usuários têm condições de acesso à telerreabilitação, é necessário destacar que os CER se concentram em capitais estaduais e municípios com maior infraestrutura^10^. Com acesso precário à internet em muitas localidades brasileiras, programas de telerreabilitação dificilmente poderiam ser aplicados de forma extensiva, e incorreriam no risco de discriminar a população excluída digitalmente caso não haja mudanças significativas nas políticas públicas^23^.

Protocolos e diretrizes para a população geral foram replicados sem personalização para PCD e seus serviços de referência^24^. As especificidades dentro deste grupo heterogêneo não foram contempladas pelos planos de enfrentamento, inclusive quanto a vulnerabilidades ainda maiores, como PCD institucionalizadas, vivendo em situação de rua, imigrantes e mulheres^25^. Tal negligência reflete um processo de invisibilidade sistemática^26^. Apesar das recomendações para a adoção de medidas inclusivas, pautadas na lógica do direito^3,27^, não se observaram dados que sugerissem essa implementação.

A maioria dos respondentes afirmou realizar algum tipo de ação voltada para o apoio social a seus usuários durante o isolamento, o que pode ser considerado positivo se comparado com a fragilidade nas ações de suporte social no enfrentamento da covid-19 identificada em outros âmbitos de assistência^28^.

A análise das políticas públicas adotadas durante o período de pandemia, assim como reformulações futuras, depende da produção de informações epidemiológicas fidedignas e específicas sobre a população que vive com deficiências. O não atendimento desse pressuposto pode configurar, por si só, um mecanismo de exclusão social, uma vez que dados desagregados sobre o acometimento pela covid-19 por status, tipo e severidade da deficiência são raros, ainda que extremamente necessários^29^.

Um dos limites deste estudo é inerente ao uso de dados do SIA-SUS. Ainda que possa haver atraso no registro dos procedimentos, trata-se de um sistema de informações com considerável agilidade e que deve ser utilizado para planejamento, análise de oferta, cobertura e seleção de prioridades^30^.

Outro limite conhecido é que o *websurvey* não utilizou uma amostra probabilística. Ainda assim, foram obtidas respostas de CER com características parecidas às dos serviços habilitados no país (Tabela 1). Acredita-se que os resultados trazem elementos importantes e contextualizam os dados de produção. As diferenças regionais existentes no país podem e devem ser objetos de novos estudos, visando identificar as especificidades locais.

Ao sobrepor duas fontes de informação, dados secundários e as percepções dos gestores, os resultados obtidos pela análise dos primeiros ganham vida, aproximando-os da realidade com o uso de abordagem metodológica pertinente ao objetivo proposto. A compreensão da realidade dos cuidados para PCD no país, incluindo territórios sem CER, e variações dentro das diferentes fases da pandemia, exigem estudos direcionados.

## CONCLUSÃO

Nos meses iniciais da pandemia de covid-19, houve um severo impacto sobre a capacidade de prestação de serviços nos CER no Brasil. É possível que tais efeitos também tenham ocorrido sobre outros serviços de reabilitação. Além da redução de procedimentos e consultas de reabilitação realizados, foram constatadas substanciais mudanças no funcionamento das equipes, que levaram ao represamento de demandas já existentes.

Os resultados evidenciam um cenário que precisa ser alterado e apontam caminhos pelos quais os formuladores de políticas e gestores de serviços podem seguir para a melhora dos cuidados às PCD.

Os serviços de reabilitação terão de lidar não apenas com a inclusão de novos protocolos de tratamento para o paciente pós-covid-19, como atender: usuários que sofreram consequências da diminuição do volume, ou da interrupção forçada, de suas terapias; os que apresentam prejuízos decorrentes de condições de saúde agravadas pelo isolamento; além de todos aqueles encaminhados por novos diagnósticos de deficiências congênitas e adquiridas. Em suma, o cenário é de extrema sobrecarga para esses serviços.

A pandemia de covid-19 ressalta a necessidade de fortalecer o SUS e a RCPD, por meio de ampliação e integração dos serviços de saúde, com a corresponsabilização entre atenção primária à saúde, atenção especializada, hospitalar e de reabilitação. O favorecimento do exercício pleno da autonomia e da capacidade funcional só poderá ser atingido a partir de proteção social ampla e organização social inclusiva, durante e após a pandemia.
